# Divergent Changes in Soil Iron-Bound Organic Carbon Between Distinct Determination Methods

**DOI:** 10.3390/biology13110852

**Published:** 2024-10-23

**Authors:** Lei Yang, Hui Yang, Ganggang Sun, Xueqin Wang, Tianli Zheng

**Affiliations:** State Key Laboratory of Herbage Improvement and Grassland Agro-Ecosystems, Lanzhou University, Lanzhou 730020, China; 220220901501@lzu.edu.cn (L.Y.); 320220901800@lzu.edu.cn (H.Y.); 13919509501@163.com (G.S.); wangxq2021@lzu.edu.cn (X.W.)

**Keywords:** soil ecosystem, soil organic carbon, iron-bound organic carbon, metal-bound organic carbon, citrate-bicarbonate-dithionite method, sulfate-dithionite method

## Abstract

Fe-OC is crucial for SOC preservation in the global ecosystem. However, there is still significant uncertainty in the determination methods of Fe-OC, and these methods are often not calibrated to each other, making the Fe-OC content by different methods impossible to compare. Here, Fe-OC is analyzed by the CBD method and the SD method from 45 soils from different land types (e.g., wetland, grassland, and forest) to compare and analyze the uncertainty and influencing factors between the two methods. Our results showed that the Fe-OC contributions to SOC (*f*Fe-OC) measured by CBD and SD methods were significantly lower in the wetland ecosystem than in grassland and forest ecosystems. The Fe-OC content and *f*Fe-OC in the grassland ecosystem was significantly higher using the CBD method compared to the SD method, with no significant difference between the methods in wetland and forest ecosystems. The random forest model revealed that Fe-OC_CBD_ content was mainly affected by C/N, Clay%, and TC, whereas SOC, total nitrogen, and soil inorganic carbon were the main influences on Fe-OC_SD_. Taken together, our findings highlight the importance of incorporating ecosystem types and soil properties into soil carbon estimation models when predicting and estimating Fe-OC and its contribution to SOC.

## Simple Summary

In this study, we analyzed the uncertainties and influencing factors associated with two different methods for determining soil iron-bound organic carbon (Fe-OC). By comparing soil samples from wetland, grassland, and forest, the results showed that the Fe-OC determined by the citrate-bicarbonate-dithionite (CBD) method (Fe-OC_CBD_) was mainly affected by the C:N ratio (C/N), clay content (Clay%), and total carbon (TC), while the Fe-OC determined by the sulfate-dithionite (SD) method (Fe-OC_SD_) was mainly affected by soil organic carbon (SOC), total nitrogen, and soil inorganic carbon. In grassland ecosystems, the choice of method significantly impacted the estimation of Fe-OC, with the CBD method proving superior to the SD method, whereas no significant differences were found between the two methods in wetlands and forests. In conclusion, future Fe-OC determinations should account for ecosystem types and soil properties, and selecting appropriate methods for different ecosystems will enhance Fe-OC estimation accuracy.

## 1. Introduction

In recent years, the role of the terrestrial ecosystem carbon cycle in predicting global climate change has garnered increasing attention [1,2,3,4,5]. Soil organic carbon (SOC) represents the largest carbon pool within the carbon cycle of terrestrial ecosystems, with its content exceeding the combined carbon stocks of vegetation and the atmosphere [6,7,8]. Notably, SOC carbon content is more than twice that of atmospheric CO_2_ and more than three-times that of terrestrial biomass carbon [3]. Therefore, even minor changes in SOC dynamics can significantly influence the terrestrial carbon cycle [5,9,10,11]. However, significant uncertainties remain regarding the processes by which SOC is formed and sequestered, complicating efforts to understand its response to global change. With the advancement of theories and research techniques, our understanding of SOC has evolved from the classical humus theory to the microbial carbon pump theory (i.e., through the in vivo turnover of microorganisms and in vitro modification of the pathway to regulate residue stabilization, leading to a renewal effect that contributes to SOC accumulation), and this has further developed into the soil mineral carbon pump theory, which emphasizes the role of microbial residues and minerals in forming mineral-bound organic carbon, thereby creating a more stable soil carbon pool [12]. The stability of SOC is strongly influenced by complex interactions between organic matter and minerals [13,14]. Therefore, gaining deeper insights into these interactions and their implications for carbon storage and sequestration is crucial for accurately predicting the impact of climate change on the global carbon cycle [1,15].

It has been established that over half of the organic carbon in the soil carbon pool is either chemically or physically bound to soil minerals [16], which affects the sequestration of organic carbon through multi-pathway coupled interactions. As a significant contributor to the stable soil carbon pool, mineral-bound organic carbon is crucial for mitigating global climate change due to its long turnover time, high stability, and low degradation rates [14,17]. Mineral-bound organic carbon comprises approximately 65% of the global mineral soil organic carbon [18,19] and possesses a longer turnover time compared to non-mineral-bound organic carbon. Thus, it plays a key role in SOC sequestration. While the mechanisms of mineral-bound organic carbon formation and preservation remain uncertain, it is now widely recognized that organic carbon and minerals form mineral–organic complexes with microbial residues, decomposition products, and soluble plant inputs through adsorption (ligand exchange, cation bridging, hydrogen bonding, and electrostatic interactions), complexation, and co-precipitation. These processes represent the primary means by which minerals sequester organic matter [20,21,22]. Evidence shows that iron oxides, owing to their large surface area and high adsorption capacity, play a crucial role in stabilizing organic carbon [23,24]. In contrast, the primary role of iron in soil is to promote the formation of soil microaggregates [25,26], acting as a cementing agent that physically protects SOC. Additionally, iron forms complexes [20] by binding to SOC through adsorption and co-precipitation, reducing the bioavailability of SOC and promoting its accumulation in soils, while also functioning as an electron acceptor or donor [27]. Notably, “rust sinks” enhance SOC stability through the formation of organo–mineral complexes between iron oxides and organic matter. In terrestrial ecosystems, the proportion of iron-bound organic carbon (Fe-OC) relative to total soil organic carbon is significantly higher in subsoils than in topsoil, with wetlands (24.5%) > grasslands (16.2%) > forests (14.9%) > farmland (14.8%) [28]. The properties and composition of soils vary among different vegetation types [29,30], leading to differing Fe-OC contents across these ecosystems. Therefore, investigating the sequestration and release of SOC by Fe-OC in diverse ecosystems is essential for forecasting the influence of climate change on the global carbon cycle. Currently, two primary methods are used for determining Fe-OC: (1) The citrate-bicarbonate-dithionite (CBD) method [31,32], which employs a neutral reducing leaching buffer of sodium citrate-bicarbonate-dithionite to reduce Fe^3+^ to Fe^2+^ in soils, ultimately extracting the Fe-oxide-fixed organic carbon. Sodium chloride is used as a control to mitigate the effects of water-soluble organic carbon, allowing for the determination of Fe-OC. However, the Fe-OC extracted using this method can be influenced by ions such as calcium (Ca^2+^) and magnesium (Mg^2+^). (2) The sulfate-dithionite (SD) method [33,34] begins with the addition of a sodium sulfate solution to eliminate the effects of water-soluble organic carbon and ions such as Ca^2+^ and Mg^2+^, followed by the addition of sodium dithionite solution to extract the Fe-OC. The differing principles of these two methods introduce various uncertainties in the determination of soil Fe-OC. As the largest organic carbon reservoir in terrestrial ecosystems, changes in soil organic carbon significantly impact atmospheric CO_2_ levels and soil fertility. This study investigates the uncertainties associated with the two primary Fe-OC determination methods in soils with different physicochemical properties across grassland, forest, and wetland ecosystems.

The objectives are (1) to determine the Fe-OC in soil using the CBD and SD methods in the three ecosystems and compare the differences between these methods; (2) to elucidate the physicochemical properties of the soil in the three ecosystems, including soil carbon, nitrogen, pH, and particle size; and (3) to analyze the results using the two methods to explore the sources of discrepancies between them.

## 2. Materials and Methods

### 2.1. Experimental Site Description

Soil samples for this study were collected from three distinct terrestrial ecosystems: wetland, grassland, and forest. For each ecosystem type, three sampling sites were selected. Grassland and wetland samples were collected from Haibei Tibetan Autonomous Prefecture (HB, 37°37′ N, 101°19′ E) in Qinghai Province, Qinghai Quanwan (QW, 36°59′ N, 99°36′ E), and Ruoergai (REG, 33°26′ N, 102°40′ E) in Sichuan Province. These sites represent alpine meadows and temperate steppes, with soils classified as chestnut, calcium, brown calcium, and meadow soils. Situated on the Tibetan Plateau, these areas range in elevation from 3194 to 3435 m and experience plateau climates, with average annual temperatures ranging from −1.70 °C to 2.90 °C and precipitation levels from 291 to 860 mm. Forest samples were collected from Suiling Forest Farm (SL, 47°40′ N, 128°04′ E) in Heilongjiang Province, Ziwuling Forest (ZWL, 36°05′ N, 108°33′ E) in Qingyang, Gansu Province, and Meiling National Forest Park (ML, 28°49′ N, 115°42′ E) in Jiangxi Province. These forests encompass cold-temperate mixed coniferous and broad-leaved forests, as well as subtropical broad-leaved evergreen forests. The soils in these areas are categorized as dark brown, meadow, loess, gray-brown, and red soils. Elevations for these sites range from 124 to 1350 m, with average annual temperatures varying from −6.00 °C to 17.60 °C, and precipitation ranging from 500 to 1950 mm (Table S1).

### 2.2. Soil Sample Collection

All soil samples were collected between June and September 2023 from three ecosystems—wetland, grassland, and forest—with three sampling sites in each ecosystem. In the wetland and grassland ecosystems, five 2 m × 2 m sample plots were selected, while five 20 m × 20 m sample plots were chosen in the forest ecosystem. Within each sample plot, five replications were randomly selected for sampling, and the five soil samples were combined to create a single composite sample. A soil auger with a diameter of 5 cm was used to collect samples from the top 0–10 cm of soil. A total of 45 soil samples were obtained across the three ecosystem types. The soil auger was thoroughly cleaned and disinfected with 75% alcohol and allowed to air-dry before sampling. The collected samples were stored in a thermostat and transported immediately to the laboratory, where they were sieved through a 2 mm mesh to remove plant roots and stones, and then air-dried naturally. These samples were then analyzed for soil pH, carbon and nitrogen content, particle size, and the concentrations of metal-bound organic carbon (Fe-OC, Ca/Mg-OC).

### 2.3. Determination of Physical and Chemical Properties of Soil

To determine soil pH, air-dried soil samples were first passed through a 2 mm sieve and then weighed into 2.00× *g* portions, adding 10 mL of ultrapure water (water–soil ratio of 1:5), and preparing the soil solution through shaking and centrifugation. The pH of the supernatant was measured using a pH meter (YSI Inc., Columbus, OH, USA), with readings taken after stabilization of the meter values. Total carbon (TC) and total nitrogen (TN) were determined from air-dried soil samples using an elemental analyzer (Thermo Fisher, Waltham, MA, USA). Soil samples that had been sieved through a 0.149 mm sieve were used to measure soil inorganic carbon (SIC) with a total organic carbon analyzer (Skalar, Breda, The Netherlands). To calculate soil organic carbon (SOC), the following equation was used:

SOC (mg g^−1^) = TC − SIC (1)

A 2.00 g portion of sieved (2 mm sieve) soil samples was treated to remove organic matter using hydrogen peroxide, followed by carbonate removal with hydrochloric acid. A suspension was prepared by adding 10 mL of a 0.50 mol L^−1^ sodium hexametaphosphate solution, and then sodium hydroxide solution was added to adjust the pH of the suspension to neutral. The mixture was allowed to stand overnight and then dispersed using an ultrasonic oscillator (160 W) for 10-to-15 min to ensure complete dispersion. The final particle size distribution was determined using a laser particle analyzer ((Microtrac, Miami, FL, USA), with results expressed as volume fractions of clay (≥0–2 μm), silt (≥2–50 μm), and sand (≥50–2000 μm).

### 2.4. Methods for Determining Iron-Bound Organic Carbon

#### 2.4.1. Iron-Bound Organic Carbon—CBD Method

The soil was first passed through a 0.25 mm sieve and then 0.50 g was weighed into a 50 mL capped centrifuge tube. Next, 30 mL of extractant A (a mixture of 0.27 mol L^−1^ trisodium citrate and 0.11 mol L^−1^ sodium bicarbonate, pH 7.3) was added, and the mixture was heated in a water bath at 80 °C for 15 min. Following this, 0.50 g of sodium dithionite powder was added, and the mixture was heated and shaken for an additional 15 min. The sample was then centrifuged at 4000 rpm for 10 min, and the supernatant was transferred to a 100 mL volumetric flask. To the solid residue, 5 mL of ultrapure water was added; the mixture was shaken well, centrifuged again, and the supernatant was added to the corresponding volumetric flask. This washing procedure was repeated five times, after which the liquid samples were fixed and preserved. The solid residue was freeze-dried, ground, and passed through a 0.149 mm sieve.

In the control experiment, 30 mL of extract B (a solution of 1.60 mol L^−1^ NaCl and 0.11 mol L^−1^ sodium bicarbonate, pH 7.3) was added to the soil samples and heated in a water bath at 80 °C for 15 min. Subsequently, 0.44 g of NaCl was added, and the mixture was heated with shaking for another 15 min. The sample was centrifuged at 4000 rpm for 10 min, and the supernatant was removed. The solid residue was then freeze-dried, ground, and passed through a 0.149 mm sieve. The organic carbon content in the residues obtained from both the CBD method and the control treatment was determined using an elemental analyzer (Thermo Fisher, USA). Prior to determination, the residue was soaked in 1 mol L^−1^ hydrochloric acid overnight to remove inorganic carbonates. After ensuring no further effervescence, the supernatant was removed, and the residue was washed five times with ultrapure water, centrifuged to remove the supernatant, oven-dried at 60 °C, and then ground again.

Fe-OC = SOC_NaCl_ − SOC_CBD_ (2)

*f*Fe-OC = Fe-OC/SOC × 100% (3)

where SOC_CBD_ and SOC_NaCl_ are the residual soil organic carbon contents after the CBD and control treatments, respectively.

#### 2.4.2. Iron-Bound Organic Carbon—SD Method

The soil was first passed through a 0.25 mm sieve and then 0.50 g was weighed into a 50 mL capped centrifuge tube. Sodium sulfate (0.50 mol L^−1^) was used for extraction at a soil-to-solution ratio of 1:5 for 1 h. The sample was then centrifuged at 4000 rpm for 20 min. The supernatant was carefully removed, and 5.00 mL of ultrapure water was added to the solid residue. The mixture was shaken well, centrifuged again, and the supernatant was discarded. This washing procedure was repeated five times to remove any remaining water-soluble organic carbon and calcium/magnesium-bound organic carbon (Ca/Mg-OC).

The residual soil was subsequently extracted with sodium dithionite (0.049 mol L^−1^) at a soil-to-solution ratio of 1:5. The suspension was vortexed for 1 min, shaken for 16 h, and then centrifuged at 4000 rpm for 20 min. The supernatant was transferred to a 100 mL volumetric flask. To the solid residue, 5 mL of ultrapure water was added; the mixture was shaken well, centrifuged, and the supernatant was added to the corresponding volumetric flask. This cleaning step was repeated five times, and the resulting liquid sample was fixed and preserved. Finally, the solution was diluted and analyzed using a total organic carbon (TOC) analyzer (Elementar vario TOC, Hanau, GER) to determine the Fe-OC content.

#### 2.4.3. Determining of Calcium/Magnesium-Bound Organic Carbon

The soil was first passed through a 0.25 mm sieve and then 0.50 g was weighed into a 50 mL capped centrifuge tube. Water-soluble organic carbon was removed by extracting the sample with ultrapure water at a soil-to-water ratio of 1:5 for 1 h. Following this, extraction with sodium sulfate (0.5 mol L^−1^) was conducted at a soil-to-solution ratio of 1:150 for 1 h. The sample was then centrifuged at 4000 rpm for 20 min. The supernatant was transferred to a 100 mL volumetric flask, and 5 mL of ultrapure water was added to the solid residue. This mixture was shaken well, centrifuged, and the supernatant was combined with the corresponding volumetric flask. The cleaning step was repeated five times to ensure complete removal of contaminants, and the liquid samples were fixed for preservation. Finally, the solution was diluted and analyzed using a total organic carbon (TOC) analyzer (Elementar vario TOC, Hanau, GER) to determine the calcium/magnesium-bound organic carbon content.

### 2.5. Statistical Analyses

All data were assessed for normal distribution and homogeneity of variance prior to conducting analysis of variance (ANOVA). Non-parametric tests were employed to compare differences in datasets that did not meet statistical assumptions, either through logarithmic transformation or using the Wilcoxon Signed-Rank Test for those that remained non-normally distributed. Differences in Fe-OC between ecosystems, as determined by the two assays, were compared using a one-way ANOVA. The correlation between soil physicochemical properties and metal-bound organic carbon was explored using the Spearman rank correlation coefficient. Random forest, a powerful and widely utilized machine learning algorithm based on decision trees, was employed for predictive modeling. Each tree within the forest provides an individual prediction, with the final output derived by averaging the results from all trees. This methodology enhances model performance and generalization. The IncNodePurity of the normalized index was utilized to assess the importance of potential predictor variables within the model [35]; a higher IncNodePurity value indicates a greater importance of the variable. Statistical analyses and data visualizations were conducted using R Studio (version 4.4.0).

## 3. Results

### 3.1. Comparison of Differences in Fe-OC and fFe-OC in Different Ecosystems

**Figure 1:**
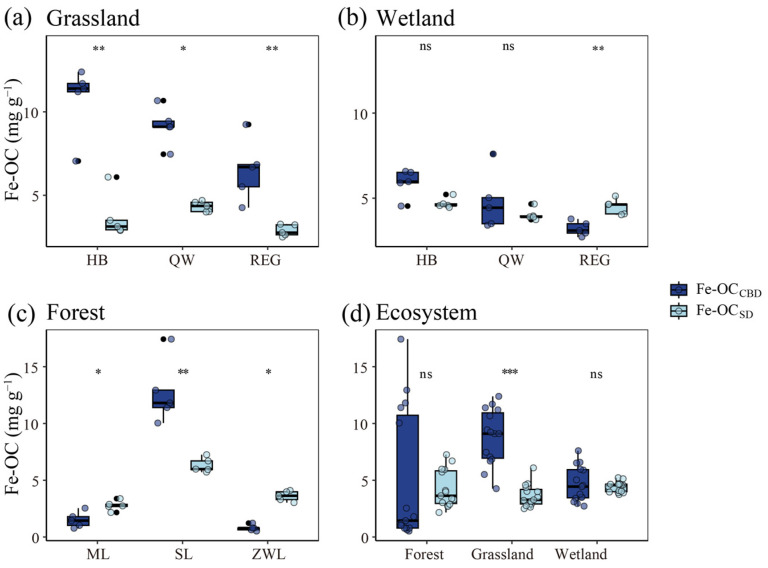
Differences in Fe-OC content extracted by the CBD and SD methods at three sample sites in grassland ecosystems, wetland ecosystems, and forest ecosystems, and among the three ecosystems. HB: the Haibei sample site, QW: the Quanwan sample site, REG: the Ruorgui sample site, ML: the Meiling sample site, SL: the Sui ling sample site, and ZWL: the Ziwuling sample site. “*” indicates significant differences at *p* < 0.05, “**” indicates significant differences at *p* < 0.01, and “***” indicates significant differences at *p* < 0.001, “ns” indicates no significant difference between. Fe-OC: the iron-bound organic carbon content. The dark blue bar represents the Fe-OC determined by the citrate-bicarbonate-dithionite (CBD) method (Fe-OC_CBD_) and the light blue bar represents the Fe-OC determined by the sulfate-dithionite (SD) method (Fe-OC_SD_).

The results among the sample sites indicated that the Fe-OC contents determined by both methods were significantly different (*p* < 0.05) in the grassland ecosystem, with the CBD method yielding significantly higher values than the SD method (Figure 1a). In the wetland ecosystems, a significant difference between the Fe-OC contents of the two methods was observed only in the REG wetland, where the SD method reported significantly higher values than the CBD method (*p* < 0.05). However, no significant differences were found in the Fe-OC contents between the two methods in the HB and QW wetlands (*p* > 0.05, Figure 1b). In forest ecosystems, the Fe-OC extracted using both the CBD and SD methods was significantly different (*p* < 0.05) across all three samples. In ML and ZWL, the Fe-OC extracted by the SD method was significantly higher than that obtained by the CBD method (*p* < 0.05), while in SL the opposite trend was observed, with the CBD method yielding significantly higher Fe-OC values than the SD method (*p* < 0.05, Figure 1c). Overall, the Fe-OC content determined by the CBD method varied from high to low across ecosystems: 8.80 ± 2.43 mg g^−1^ in grassland, 5.01 ± 5.87 mg g^−1^ in forest, and 4.64 ± 1.54 mg g^−1^ in wetland. In contrast, the Fe-OC values obtained using the SD method were 4.41 ± 0.46 mg g^−1^ in wetland, 4.23 ± 1.64 mg g^−1^ in forest, and 3.64 ± 0.98 mg g^−1^ in grassland (Table S2). In grassland ecosystems, the difference in Fe-OC content between the two methods was highly significant, with the CBD method yielding values significantly higher than those from the SD method (*p* < 0.001). In contrast, no significant differences in Fe-OC content were observed between the two methods in wetland and forest ecosystems (*p* > 0.05, Figure 1d).

**Figure 2:**
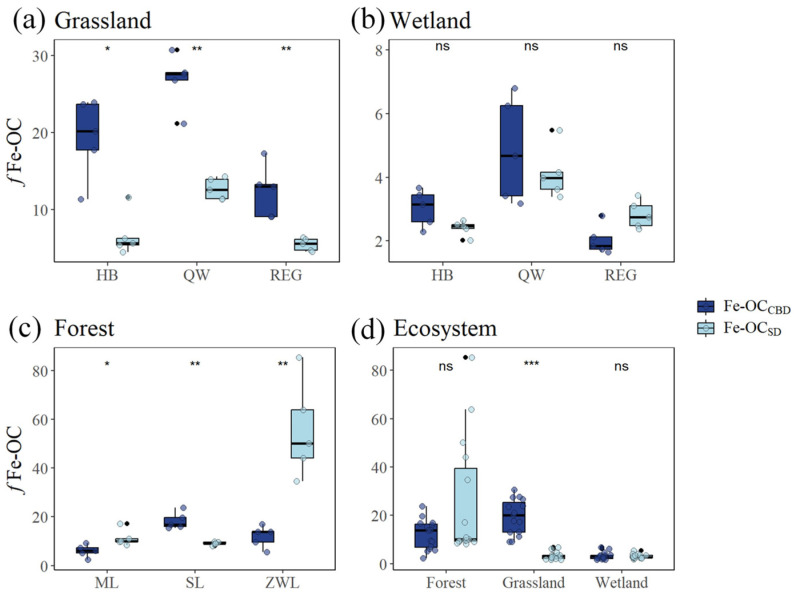
Differences in *f*Fe-OC content extracted by the CBD and SD methods at three sampling sites in grassland, wetland, and forest ecosystems, and among the three ecosystems. *f*Fe-OC: the Fe-OC contribution to SOC. “*” indicates significant differences at *p* < 0.05, “**” indicates significant differences at *p* < 0.01, and “***” indicates significant differences at *p* < 0.001, “ns” indicates no significant difference between.

Comparison of the two methods across different ecosystems revealed that the Fe-OC contribution to SOC (*f*Fe-OC) determined by the CBD method ranged from high to low: 19.49 ± 7.21% in grassland, 12.15 ± 6.16% in forest, and 3.31 ± 1.55% in wetland. In contrast, the *f*Fe-OC determined by the SD method showed a different order, with values of 25.35 ± 24.61% in forest, 8.28 ± 3.70% in grassland, and 3.12 ± 0.91% in wetland (Table S2). The significant difference in *f*Fe-OC was observed between the two methods in grassland ecosystems, where the CBD method yielded significantly higher values than the SD method. However, no significant differences in *f*Fe-OC were found between the two methods in wetland and forest ecosystems (Figure 2d). Among the ecosystems, the *f*Fe-OC determined by the two methods varied significantly among the three sample sites in grassland ecosystems, with the CBD method exhibiting significantly higher values than the SD method (*p* < 0.05, Figure 2a). In wetland ecosystems, the *f*Fe-OC determined by the two methods did not differ significantly among the three sample sites (Figure 2b). In forest ecosystems, the *f*Fe-OC values determined by the two methods were significantly different across the three sample sites, with the SD method yielding significantly higher values in Meiling and ZWL forests, while the CBD method was significantly higher in the SL forest (*p* < 0.05, Figure 2c).

### 3.2. Determination of Fe-OC by the Two Methods and the Relationship Between fFe-OCs

**Figure 3:**
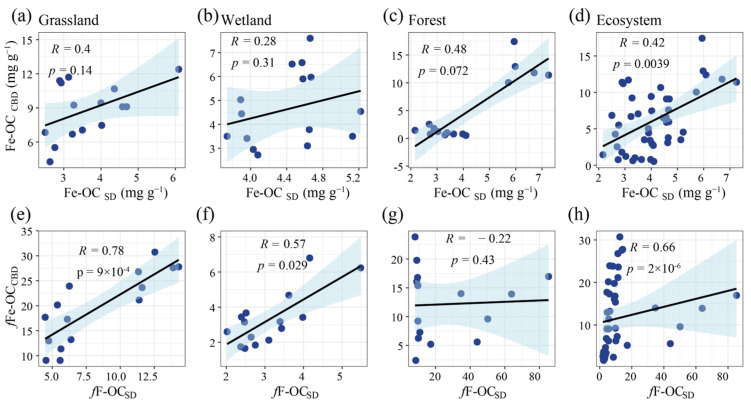
(**a**–**c**,**e**–**g**) show the correlation analyses between Fe-OC and *f*Fe-OC determined by CBD and SD methods in grassland ecosystems, wetland ecosystems, and forest ecosystems, respectively; figures (**d**,**h**) show the correlation analyses between Fe-OC and *f*Fe-OC determined by CBD and SD methods among all ecosystems. *f*Fe-OC_CBD_: the Fe-OC, as determined by the citrate-bicarbonate-dithionite (CBD) method, contribution to SOC, *f*Fe-OC_SD_: the Fe-OC, as determined by the sulfate-dithionite (SD) method, contribution to SOC.

The results of Fe-OC in different ecosystems showed a significant positive correlation between the Fe-OC content in the soil determined by the CBD and SD methods (*R* = 0.42, *p* < 0.01, Figure 3d). However, within individual ecosystems, no significant relationship was observed between the Fe-OC content determined by the two methods (Figure 3a–c). The results of *f*Fe-OC in different ecosystems showed a significant positive correlation between *f*Fe-OC in soil determined by the CBD and SD methods (*R* = 0.66, *p* < 0.001, Figure 3h). The results of *f*Fe-OC between the sample sites of each ecosystem showed a significant positive correlation between *f*Fe-OC in soil determined by the CBD and SD methods in grassland and wetland ecosystems (*p* < 0.001, Figure 3e,f), while in forest ecosystems no significant relationship was observed (Figure 3g).

### 3.3. Factors Affecting Fe-OC and fFe-OC

**Figure 4:**
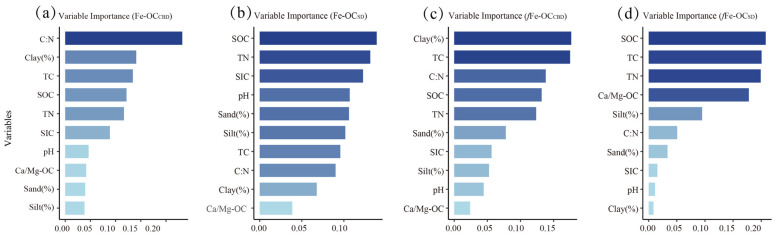
The relative importance of the factors influencing Fe-OC and *f*Fe-OC was determined by the CBD and SD methods using the random forest model. The darker blue color means the more important factor. SOC: soil organic carbon; C/N: C:N ratio; Clay%: clay content; Silt%: silt content; Sand%: sand content; TC: total carbon; TN: total nitrogen; SIC: soil inorganic carbon; Ca/Mg-OC: calcium/magnesium-bound organic carbon.

The results of the random forest model showed that the most important factor influencing the extraction of Fe-OC by the CBD method (Fe-OC_CBD_) was the C/N, with a 23.2% importance among all factors, followed by Clay% and TC with a 14.1% and 13.4% importance, respectively (Figure 4a). The most important factors influencing the extraction of Fe-OC by the SD method (Fe-OC_SD_) were SOC, TN, and SIC, with a 13.9%, 13.1%, and 12.3% importance, respectively (Figure 4b). The most important factors influencing Fe-OC_CBD_ contribution to SOC are Clay%, TN, and C/N, with importance shares of 17.6%, 17.5%, and 13.8%, respectively (Figure 4c). The most important factors influencing the Fe-OC_SD_ contribution to SOC were SOC, TC, TN, and Ca/Mg-OC, with importance percentages of 20.8%, 20.1%, 19.9%, and 17.8%, respectively (Figure 4d).

## 4. Discussion

### 4.1. Differences in Fe-OC and fFe-OC in Different Ecosystems

In this study, significant differences were observed in the Fe-OC content measured by the CBD and SD methods at most of the sample sites (Figure 1). In grassland ecosystems, the Fe-OC content measured using the CBD method was significantly higher (*p* < 0.05) than that obtained with the SD method. This discrepancy is likely attributable to the inherent nature of the CBD method, which employs a strong reductive extraction [34], potentially releasing more Fe-OC than the SD method. By examining the principles of both methods, we posit that the CBD method extracts Fe-OC by also releasing other metals bound to organic carbon, resulting in a higher Fe-OC content compared to the SD method. However, we found that the content of Fe-OC extracted by the SD method, together with Ca/Mg-OC, remained significantly lower than that of the Fe-OC extracted by the CBD method (*p* < 0.05, Figure S1). The significantly lower Fe-OC extracted by the SD method compared to the CBD method is likely due to the incomplete extraction of Fe-OC by the SD method. Grasslands are estimated to contribute 121 Mt C yr^−1^ of Fe-OC burial in terrestrial ecosystems [36]. Thus, the higher Fe-OC content in grassland ecosystems is associated with enhanced SOC stabilization. Soil physicochemical properties, such as pH and C/N, significantly influence Fe-OC content across ecosystems [37] (Figure S2). A low soil pH was found to increase Fe extraction but hinder the release of Fe-OC, suggesting that higher pH levels in grassland soils may enhance the efficacy of the CBD method for Fe-OC extraction [38,39]. In forest ecosystems, the difference in Fe-OC content measured by the CBD and SD methods was not statistically significant (*p* > 0.05). However, site-specific variations were noted: the SD method extracted more Fe-OC in the Meiling and ZWL forests, while the CBD method was more effective in the SL forest. Differences in soil properties, particularly texture and organic matter content, are likely the primary factors influencing these variations [40,41]. In wetland ecosystems, more Fe-OC was extracted by the SD method than by the CBD method in the REG wetland. This is likely due to the unique composition of wetland soils, which typically contain a higher organic matter content and more diverse forms of iron oxides [42,43,44]. Overall, the higher soil Fe oxide content in grassland ecosystems enhances the CBD method’s capacity to extract Fe-OC by reducing Fe^3+^ to Fe^2+^ with a strong reductant. Conversely, the higher organic matter content and complex morphology of iron oxides in wetland soils reduce the difference in extraction results between the two methods [45,46,47]. Therefore, the varying soil properties and compositions in different vegetation types lead to distinct Fe-OC contents across ecosystems (Figure S3). The CBD method proved more effective in grasslands, while the SD method was more proficient in specific forest and wetland contexts. These findings underscore the importance of selecting an appropriate extraction method tailored to soil properties and ecosystem types for accurate Fe-OC measurement.

### 4.2. Relationship Between Fe-OC and fFe-OC Determined by Different Methods

The Fe-OC content determined by the CBD and SD methods was significantly correlated across all ecosystems (*R* = 0.42, *p* < 0.01, Figure 3d), suggesting that both methods are generally reliable for Fe-OC extraction, despite differences in extraction efficiency by soil type. This correlation, however, was not observed within individual ecosystems, indicating that soil-specific factors significantly influence extraction efficiency and the suitability of each method. *f*Fe-OC results exhibited a stronger correlation across ecosystems (*R* = 0.66, *p* < 0.001, Figure 3h), with significant positive correlations in both grassland and wetland ecosystems, further supporting the consistency of the two methods in these settings. In contrast, the correlation in forest ecosystems was not significant, likely due to complex interactions between organic matter and iron oxides, which result in variable extraction outcomes [48]. According to a recent meta-analysis [28], *f*Fe-OC levels in grassland, wetland, and forest ecosystems were estimated to be 16.2 ± 11.4%, 24.5 ± 19.3%, and 14.9 ± 10.5%, respectively. In comparison, our study suggests that the SD method may substantially underestimate Fe-OC content in grasslands, potentially by nearly half. In wetland ecosystems, the values determined by both methods were significantly lower than those reported in the meta-analysis, likely due to the fact that our soil samples were taken from flooded wetlands, which inhibited co-precipitation processes and led to lower Fe-OC content [49]. Moreover, in forest ecosystems the SD method was shown to underestimate Fe-OC content by more than 70%, highlighting the importance of method selection for accurate Fe-OC measurement. Therefore, selecting appropriate methods for Fe-OC content determination is essential for improving predictions of soil carbon–climate feedbacks at national and global scales.

### 4.3. Factors Affecting Fe-OC and fFe-OC

The random forest model revealed that various soil properties significantly influenced Fe-OC extraction by both the CBD and SD methods. For the CBD method, the most influential factor was the C/N (23.2%), followed by Clay% and TC. The significant negative correlation between Fe-OC and the C/N (*R* = −0.79, *p* < 0.001, Figure S4e) indicates that soils with a higher C/N inhibit Fe-OC extraction. In contrast, the significant positive correlation between Fe-OC_CBD_ and Clay% (*p* < 0.05, Figure S4h) suggests that a higher Clay% enhances Fe-OC extraction content. Clay minerals promote iron oxide–carbon binding through adsorption and covalent interactions, while the small pores in clay-rich soils limit microbial degradation and enhance Fe-OC stability [50]. The positive correlation between TC and Fe-OC_CBD_ (Figure S4c) indicated that soils with a higher TC reveal a higher extraction of content using the CBD method due to stronger binding between iron oxides and organic matter [51]. For the SD method, SOC, TN, and SIC were the most influential factors, and the significant positive correlations between Fe-OC_SD_ and SOC, as well as TN, align with the random forest model results. The SD method’s efficiency seems to be influenced by a higher carbon content, likely due to its milder extraction conditions that preferentially release Fe-OC from soils with a higher carbon content. Clay% and TN were the most important factors influencing the CBD method, while SOC, TC, TN, and Clay% were crucial for the SD method (Figures S4 and S5). These findings highlight the complex interplay between soil physical and chemical properties in determining Fe-OC extraction efficiency and their contribution (Figures S6 and S7) to SOC stabilization [26,36].

Differences in Fe-OC content and influencing factors across methods and ecosystems suggest that Fe-OC extraction methods should be selected based on specific soil properties and ecosystem types. Understanding these differences is essential for accurately assessing Fe-OC and its role in soil carbon stabilization. The higher Fe-OC content observed in grasslands using the CBD method suggests possible interference by Ca^2+^/Mg^2+^, which could affect carbon sequestration through iron oxides. In contrast, the SD method more accurately determines Fe-OC by removing Ca^2+^/Mg^2+^ and water-soluble organic carbon, though it may have limitations in soils with a high organic matter content. The SD method may be more suitable for forest and wetland ecosystems, where the interaction between organic matter and iron oxides is more complex. The results of this study are critical for predicting carbon cycle dynamics and understanding the role of Fe-OC in soil carbon stocks under varying land-use and climatic conditions. Accurate Fe-OC measurements are essential for evaluating its contribution to long-term carbon sequestration and potential feedback to global climate change.

## 5. Conclusions

In this study, we compared the CBD and SD methods for determining Fe-OC and *f*Fe-OC across grassland, wetland, and forest ecosystems, revealing their applicability and sources of uncertainty. The results indicated that the CBD method was more effective than the SD method for determining Fe-OC in grassland ecosystems, while no significant differences were observed between the methods in wetland and forest ecosystems. Fe-OC determination using different methods was significantly influenced by soil physicochemical properties, and the random forest model demonstrated that each method was impacted by distinct soil factors. To improve the accuracy of Fe-OC determination, future studies should integrate multiple methods and consider soil physicochemical properties holistically. Additionally, the efficiency and precision of Fe-OC extraction could be enhanced by refining existing methods and minimizing interfering factors during the determination process. Combining these findings with data from specific ecosystems will deepen our understanding of soil carbon dynamics and provide a scientific basis for optimizing land management to maximize carbon sequestration. Further research should explore the formation mechanisms and stability of Fe-OC in various ecosystems to better understand the dynamics of soil organic carbon pools and sequestration mechanisms. Overall, our study underscores the complexity of Fe-OC measurements and highlights the need for a nuanced approach that considers soil properties, ecosystem types, and methodological differences to accurately assess its role in soil carbon sequestration and climate change mitigation.

## Data Availability

All data from this study are included in the article and Supplementary Materials.

## Supplementary Materials

The following supporting information can be downloaded at: https://www.mdpi.com/article/10.3390/biology13110852/s1, Figure S1: Differences in extracted Fe-OC content between Fe-OC_CBD_ and Fe-OC_SD_ + Ca/Mg-OC at three sample sites in grassland ecosystems, wetland ecosystems and forest ecosystems and among the three ecosystems; Figure S2: Heat map analysis of correlations between all soil factors; Figure S3: Distribution of Clay (%), Silt (%) and Sand (%) content of the soil, with colors added to the sample points in the a, b, c and d plots according to the high and low values of Fe-OC_CBD_, Fe-OC_SD_, *f*Fe-OC_CBD_, *f*Fe-OC_SD_ content, respectively; Figure S4: Correlation analysis between Fe-OC_CBD_ and SOC, SIC, TC, TN, C/N, Ca/Mg-OC, pH, Clay (%), Silt (%) and Sand (%); Figure S5: Correlation analysis between Fe-OC_SD_ and SOC, SIC, TC, TN, C/N, Ca/Mg-OC, pH, Clay (%), Silt (%) and Sand (%); Figure S6: Correlation analysis between *f*Fe-OC_CBD_ and SOC, SIC, TC, TN, C/N, Ca/Mg-OC, pH, Clay (%), Silt (%) and Sand (%); Figure S7: Correlation analysis between *f*Fe-OC_SD_ and SOC, SIC, TC, TN, C/N, Ca/Mg-OC, pH, Clay (%), Silt (%) and Sand (%); Table S1: Latitude, longitude, elevation, mean annual temperature, annual precipitation, soil type and dominant species of different ecosystem sampling sites; Table S2: Fe-OC and *f*Fe-OC measurements; Table S3: Measurement results of soil properties.

## Abbreviations

Fe-OC: iron-bound organic carbon; *f*Fe-OC: Fe-OC contribution to SOC; CBD: citrate-bicarbonate-dithionite; SD: sulfate-dithionite; SOC: soil organic carbon; C/N: C:N ratio; Clay%: clay content; Silt%: silt content; Sand%: sand content; TC: total carbon; TN: total nitrogen; SIC: soil inorganic carbon.
