# Supplementary material for: Divergent Changes in Soil Iron-Bound Organic Carbon Between Distinct Determination Methods
Source: Biology (Basel). 2024 Oct 23;13(11):852. doi: 10.3390/biology13110852 (PMC11592089; doi:10.3390/biology13110852)
Supplement: Supplementary file 1 [file biology-13-00852-s001.zip › biology-3208951-supplementary.pdf]

Supplementary Materials for

Divergent Changes of Soil Iron-bound Organic Carbon between Distinct Determination Method

Lei Yang et al.

Corresponding author: Tianli Zheng, zhengtl@lzu.edu.cn

The PDF file includes:

Figure. S1 to S7

Tables S1 to S3

Table S1: Latitude, longitude, elevation, mean annual temperature, annual precipitation, soil type and dominant species of different ecosystem sampling sites

Ecosystem type	Site	Lon & lat	Climate zone	DEM (m)	MAT (℃)	MAP (mm)	Soil type[1]	Dominant species
Grassland	REG	102°40′E, 33°26′N	Highland cold humid monsoon climate	3435	2.05	683	meadow soil	*Leontopodium leontopodioides* *Kobresia tibetica*、*Blysmus sinocompressus*、
	QW	99°36′E, 36°59′N	Semi-arid climate of the inland plateau	3194	0.38	305	calcium chestnut soil	*Poa pratensis* *Agropyron cristatum*、*Elymus nutans*、
	HB	101°19′E, 37°37′N	highland continental climate	3244	-0.71	468	Brown Calcium Soil Chestnut Calcium Soil,	*Elymus nutans*、*Poa spp.*、*Stipa aliena*
Wetland	REG	102°40′E, 33°26′N	Highland cold humid monsoon climate	3435	2.05	683	Boggy soil, peat soil	*Potentilla anserina* *Carex muliensis*、*C.meyerina*、
	QW	99°36′E, 36°59′N	Semi-arid climate of the inland plateau	3194	0.38	305	Boggy soil	*Lobularia maritima* *Kobresia humilis*、*Carex tristachya*、
	HB	101°19′E, 37°37′N	highland continental climate	3244	-0.71	468	Boggy soil, peat soil	*Blysmus sinocompressus* *Kobresia tibetica*、*Carex pamirensis*、
Forest	ML	115°42′E, 28°49′N	subtropical monsoon climate	124	16.55	1574	red soil	*Pinus massoniana Lamb.*、 *Cunninghamia lanceolata (Lamb.) Hook.*、
	ZWL	108°33′E, 36°05′N	semi-arid monsoon climate	1350	8.54	509	Loess, gray-brown soil	*Quercus mongolica*、*Pinus tabuliformis*
	SL	128°04′E, 47°40′N	temperate continental climate	278	0.94	599	meadow soil Dark brown loam,	*Betula platyphyllaSuk.* *Abies fabri Craib*、*Picea koraiensis Nakai*、

[1]Note: Soil classification is based on the Chinese soil taxonomy

Table S2: Fe-OC and *f*Fe-OC measurements

Ecosystem type	Site	Fe-OC$_{CBD}$ (mg g^{-1})	Fe-OC$_{SD}$ (mg g^{-1})	*f*Fe-OC$_{CBD}$ (%)	*f*Fe-OC$_{SD}$ (%)
Grassland	HB	10.75±2.12	3.71±1.35	19.35±5.16	6.67±2.83
	QW	9.15±1.15	4.33±0.32	26.81±3.5	12.69±1.37
	REG	6.51±1.85	2.88±0.35	12.32±3.43	5.47±0.81
	Grassland Mean	8.8±2.43	3.64±0.98	19.49±7.21	8.28±3.7
Wetland	HB	5.9±0.82	4.7±0.3	3.03±0.57	2.4±0.23
	QW	4.8±1.71	4.03±0.36	4.87±1.63	4.13±0.81
	REG	3.21±0.43	4.5±0.45	2.03±0.46	2.83±0.44
	Wetland Mean	4.64±1.54	4.41±0.46	3.31±1.55	3.12±0.91
Forest	SL	12.72±2.83	6.32±0.64	18.37±3.48	9.14±0.64
	ZWL	0.79±0.27	3.61±0.45	12.01±4.45	55.62±19.7
	ML	1.51±0.69	2.77±0.43	6.07±2.53	11.3±3.41
	Forest Mean	5.01±5.87	4.23±1.64	12.15±6.16	25.35±24.61

Table S3: Measurement results of soil properties

Ecosystem-type	Site	SOC (mg g^{-1})	SIC (mg g^{-1})	TC (mg g^{-1})	TN (mg g^{-1})	C/N	Ca/Mg-OC (mg g^{-1})	pH	Clay (%)	Silt (%)	Sand (%)
Grassland	HB	56.74±7.16	2.95±0.28	59.69±7.4	5.3±0.72	10.72±0.27	1.25±0.85	8.18±0.21	4.35±2.17	57.77±18.83	37.88±20.97
	QW	34.2±1.23	13.33±0.9	47.53±1.36	2.8±0.13	12.24±0.52	1.04±0.46	7.55±0.03	4.96±1.23	68.09±12.38	26.95±13.59
	REG	52.91±5.06	13.63±1.25	66.54±4.4	4.84±0.44	10.92±0.18	0.81±0.24	7.71±0.06	6.33±0.42	83.6±3.4	10.07±3.64
	Mean	47.95±11.24	9.97±5.21	57.92±9.37	4.31±1.21	11.29±0.77	1.03±0.56	7.81±0.3	5.21±1.6	69.82±16.41	24.97±17.96
Wetland	HB	196.58±18.46	14.94±11.86	211.52±12.93	14.97±1.2	13.17±1.17	1.3±0.23	6.84±0.47	0.57±0.15	45.69±3.26	53.74±3.34
	QW	99.99±16.8	18.5±1.32	118.49±15.94	7.85±1.63	12.87±0.82	0.75±0.24	7.58±0.09	0.63±0.21	45.56±5.94	53.81±6.13
	REG	160.72±14.25	19.8±5.02	180.52±15.75	11.56±1.08	13.91±0.27	0.34±0.17	7.34±0.13	0.38±0.04	34.69±4.43	64.93±4.41
	Mean	152.43±44.03	17.75±7.24	170.18±42.36	11.46±3.25	13.31±0.9	0.79±0.45	7.25±0.42	0.53±0.18	41.98±6.87	57.49±7.01
Forest	SH	69.2±6.34	0.16±0.03	69.36±6.36	5.93±0.63	11.69±0.28	0.72±0.13	6.26±0.07	4.78±1.72	62.23±5.42	32.98±6.71
	QY	6.99±1.98	10.62±3.23	17.6±1.26	0.37±0.16	19.76±3.6	0±0	7.25±0.14	2.62±0.32	87.59±2	9.79±2.11
	ML	25.53±5.57	0.15±0.01	25.67±5.57	1.32±0.36	19.66±1.61	1.23±0.28	4.37±0.24	3.15±1.84	62.74±9.58	34.11±11.01
	Mean	33.9±27.39	3.64±5.39	37.55±23.98	2.54±2.54	17.04±4.45	0.65±0.55	5.96±1.25	3.52±1.66	70.85±13.63	25.63±13.54

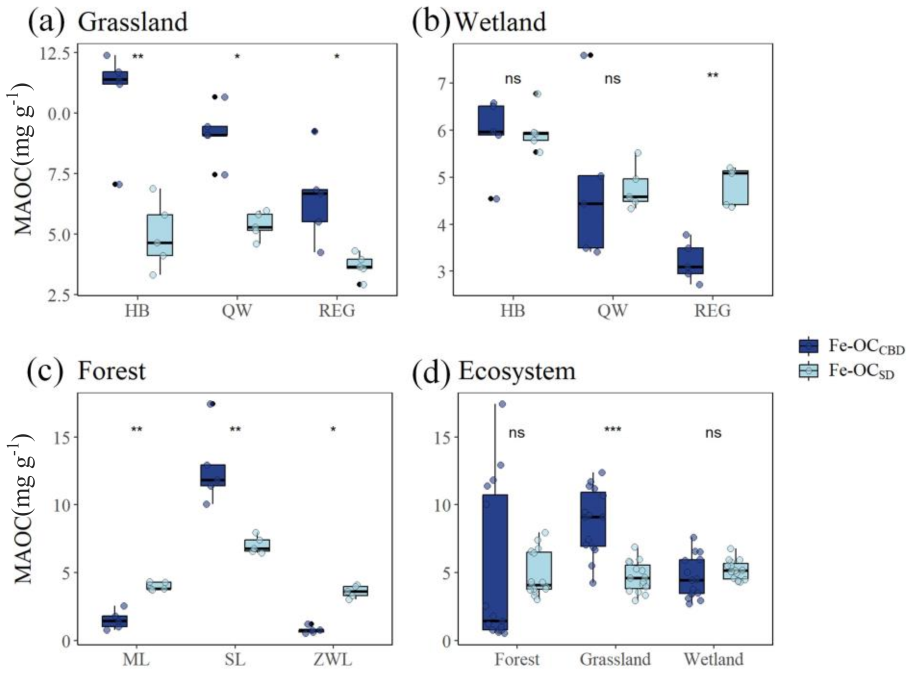

Figure S1: Differences in extracted Fe-OC content between Fe-OC$_{CBD}$ and Fe-OC$_{SD}$ + Ca/Mg-OC at three sample sites in grassland ecosystems, wetland ecosystems and forest ecosystems and among the three ecosystems

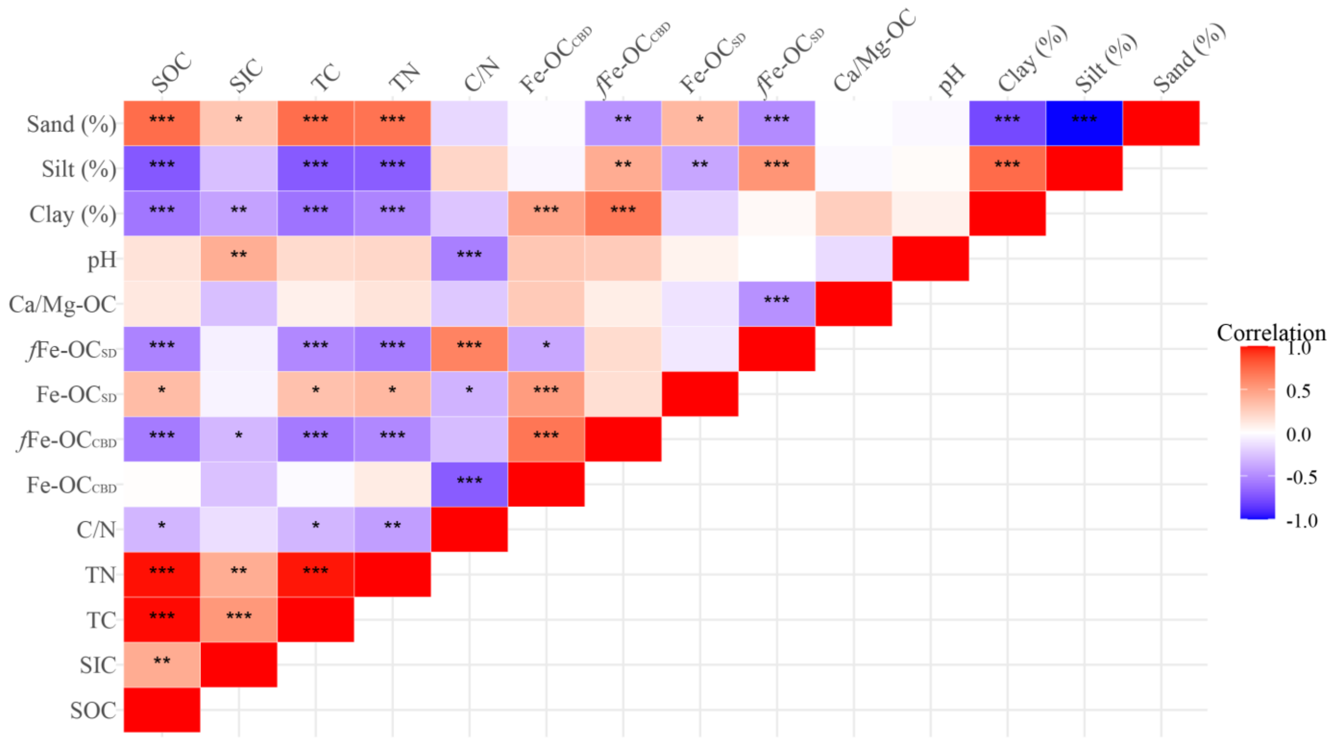

Figure S2: Heat map analysis of correlations between all soil factors

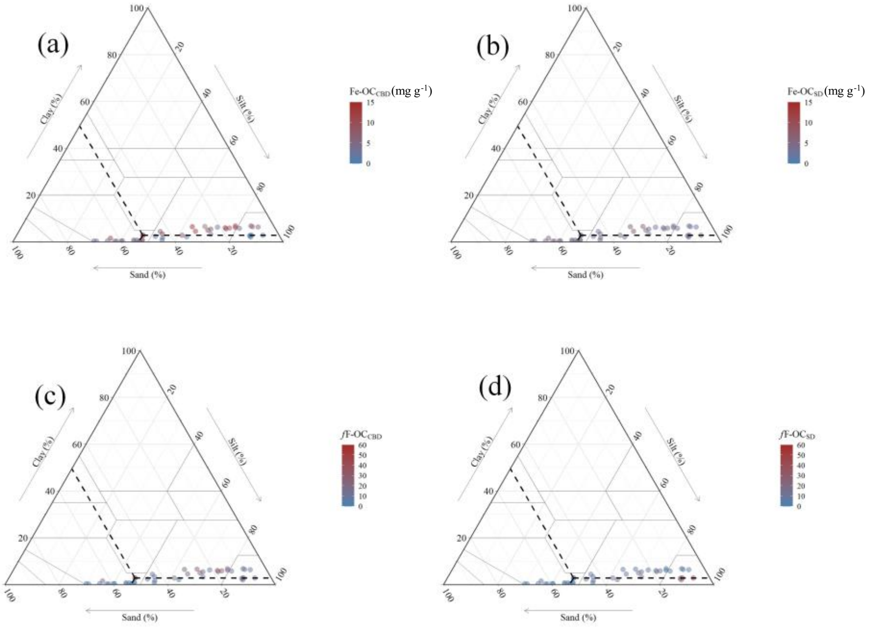

Figure S3: Distribution of clay (%), silt (%) and sand (%) content of the soil, with colors added to the sample points in the a, b, c and d plots according to the high and low values of Fe-OC$_{CBD}$, Fe-OC$_{SD}$, fFe-OC$_{CBD}$, fFe-OC$_{SD}$ content, respectively

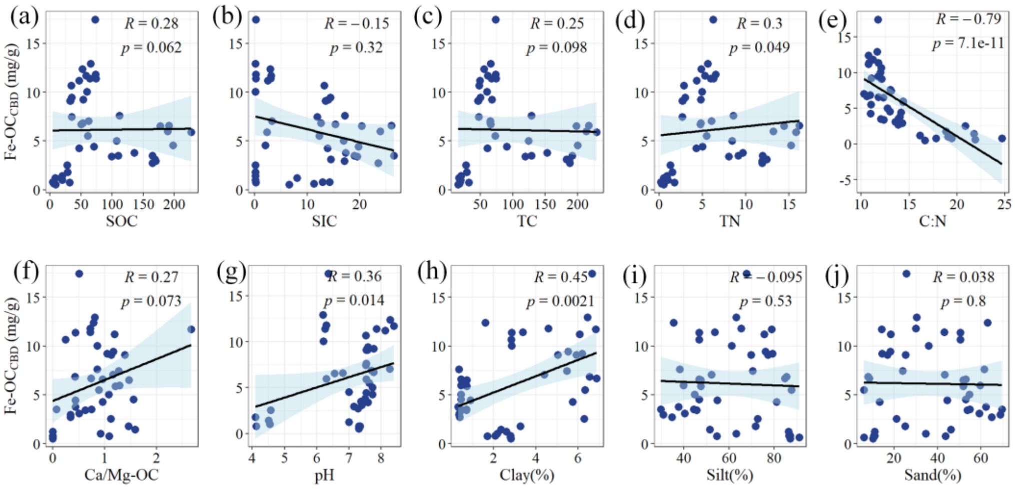

Figure S4: Correlation analysis between Fe-OC$_{CBD}$ and SOC, SIC, TC, TN, C/N, Ca/Mg-OC, pH, clay (%), silt (%) and sand (%)

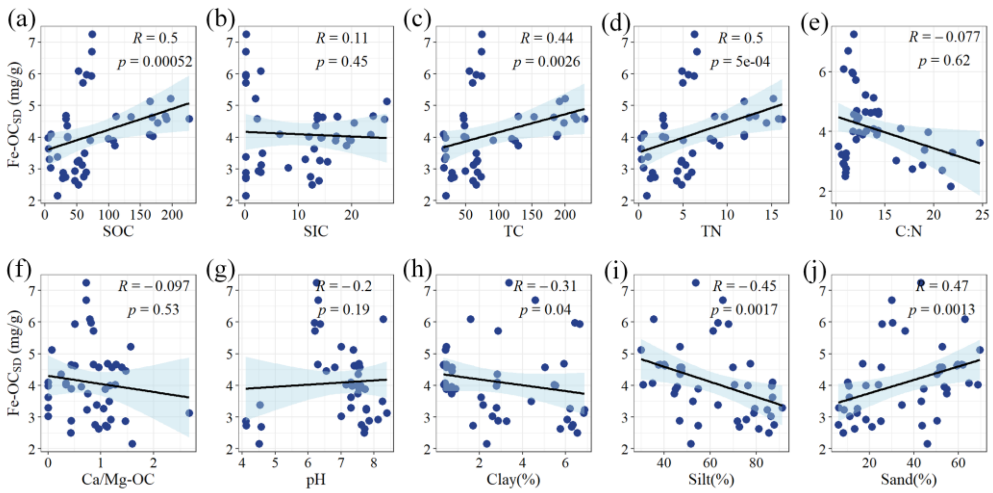

Figure S5: Correlation analysis between Fe-OC$_{SD}$ and SOC, SIC, TC, TN, C/N, Ca/Mg-OC, pH, clay (%), silt (%) and sand (%)

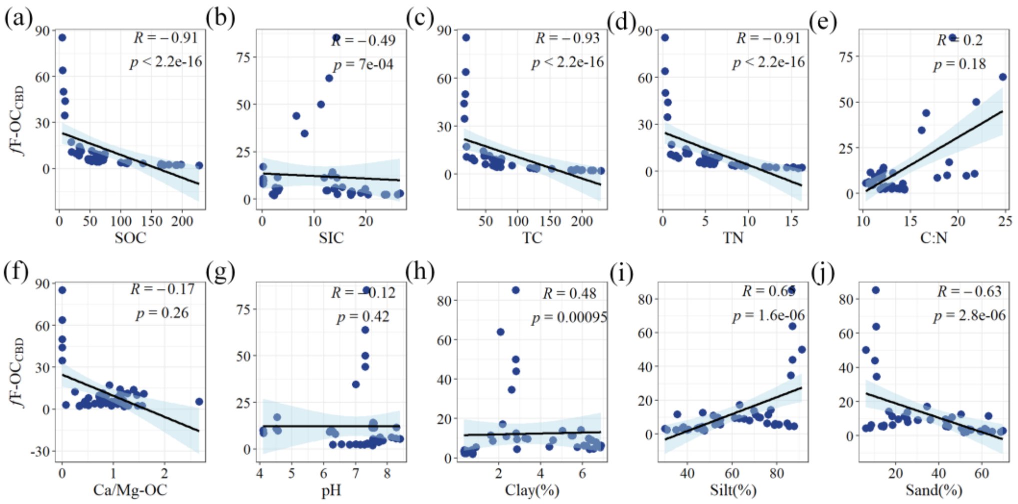

0

Figure S6: Correlation analysis between *f*Fe-OC$_{CBD}$ and SOC, SIC, TC, TN, C/N, Ca/Mg-OC, pH, clay (%), silt (%) and sand (%)

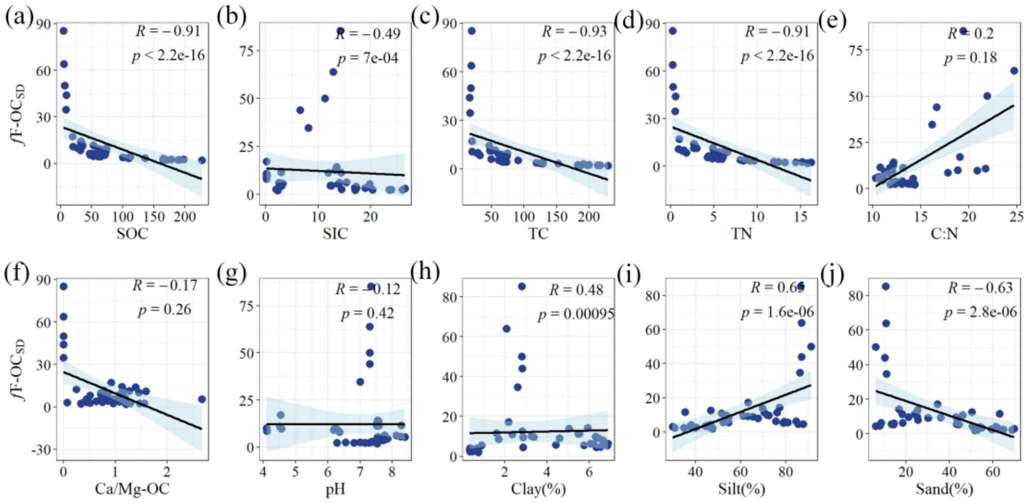

Figure S7: Correlation analysis between *f*Fe-OC$_{SD}$ and SOC, SIC, TC, TN, C/N, Ca/Mg-OC, pH, clay (%), silt (%) and sand (%)
